# Some Surgical Complications Following Influenza

**Published:** 1915-02

**Authors:** W. L. Crosthwait


### TEXAS MEDICAL JOURNAL

**Dr. F. E. Daniel, Founder. Established July, 1885**

MRS. F. E. DANIEL, - Publisher and Managing Editor

Published Monthly.—Subscription, $1.00 a Year.

VOL. XXX AUSTIN, FEBRUARY, 1915 No. 8

**The publisher is not responsible for views of contributors**

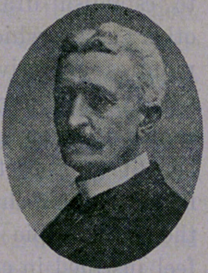

### Original Articles.

### Some Surgical Complications Following Influenza.

BY W. L. CROSTHWAIT.

It is scarcely within the scope of a short paper to discuss all of the surgical complications following infections due to the small bacilli of Pfeiffer, or the influenza bacilli. Neither is it possible to deal with the minute pathology and operative technic of even a few of the more common surgical complications and sequelae.

The bacilli of Pfeiffer, as we understand it, is aerobic, and a facultative pus producer. Its love for company or accomplices in devilment is one of its marked characteristics. It seems to have a special affinity for the staphylococcus and pneumococcus, and it is not averse to associating itself with the streptococcus. Its virulence is greatly increased by association with these various micro-organisms. Not only does it increase in size and number, but the toxins liberated when the bacilli are split up seem to be greatly augmented. Furthermore, this bacillus has the faculty of selecting its field of activity in certain tissues and membranes of the body where various other organisms are most liable to hibernate or invade, namely, the frontal sinus and accessory sinuses, the antrum of Highmore, the middle ear, the mastoid cells, also the pleura and peritoneum. The most common surgical lesions resulting as complications or sequelae to la grippe are, therefore suppurative conditions due to infection of these parts. Following my own conclusion and observation, and I am frank to say that I have no authority to support me in this conclusion, I mention as the first and most common infection that of the frontal sinus or a frontal sinusitis. I have seen this complication result in quite a number of cases, five of which required operative treatment to establish drainage, and this list does not inclube probably other cases which lapsed into the chronic stage, which later requires operative treatment, at the hands of the specialist to whom they were referred. Some of these cases occurred at a time when I could not avail myself of the diagnostic benefit of a bacteriological laboratory, but they were so similar in all of their characteristics to the cases occurring later, in which the microscopic examinations made plain the diagnosis, that I feel justified in concluding that all belonged to the same class.

The next most common surgical complications are infections of the antrum. Quite a number of such cases have occurred under my observation. Of course I have never observed a suppurative condition of the antrum that was not a distinct mixed infection.

The third line that characterizes surgical lesions following la grippe are infections of the mastoid cells, in other words acute mastoiditis. This is always a most serious condition and is distinctly a surgical disease. I wish to report briefly a case coming under my observation in which repeated microscopical examinations showed an almost pure Pfeiffer bacillus infection, and this was one of the most virulent and obstinate infections I have ever seen. This infection not only followed the lymphatic channel, but seemed to have a special affinity for the fascia, and before it was finally controlled it had destroyed the posterior and anterior auricular lymphatic nodes, parotid ganlds, paridio masseteric fascia. When incising for the purpose of drainage the scalpel reached the periosteum over the squamos portion of the temporal bone, resulting in infection of periosteum, which spread over a large area. When this case came into my hands a superficial opening had been made to drain the posterior cervical lymphatics. There was an active suppurative process over the mastoid process with necrosis of tone tissue requiring curetment or radical removal of all the mastoid process which was swabbed out with a tincture of iodine. A long incision was made along the sterno-cleido mastoid muscle. The mastoid trouble healed very rapidly, the suppuration disappearing within a few days and the patient returned home. She returned later with infection, which had spread upon the anterior auricular node of lymphatic, involving the parotid gland. The face was greatly swollen. Free incision was made and large quantities of pus evacuated and drainage established. This pus showed a few staphylococcus, but a great abundance of bacilli Pfeiffer were shown. At the end of two weeks the patient again left the hospital. Three days later she returned with a tumefaction over the squamos portion of the temporal bone. This was opened, pus evacuated and drainage established. In making this incision, thinking that the infections perhaps extended down to the periosteum, a deep incision was made and the periosteum injured. Within two days the infection spread over the area bordered anteriorly by the frontalis muscle fascia attachment and posteriorly by the occipatalis. In fact, the infection was apparently co-extensive with the superficial fascia which was incised for drainage evacuating a large quantity of pus. The cavity was sponged out thoroughly with tincture of iodine, twice daily for two days, after which the infection was under control. This pus gave the same findings as previously. This began as a case of la grippe, followed by otitis media, which was treated in the usual way. I may be mistaken, but I am strongly inclined to believe the bacillus of Pfeiffer was responsible for practically all of the suppurative lesions connected with this case.

The next most common surgical complication following la grippe is empyema. I have never observed a case of empyema that was not also a mixed infection. But I recall one case in which I made repeated microscopical examination and demonstrated the bacilli of Pfeiffer as being conspicuous and predominant for their constant appearance. Peritonitis due to infections directly or indirectly of bacilli of Pfeiffer are reported by a number of men. The case which I have observed seemed to result from extension of pus activity which seems to be a sequela or complication of la grippe, as autopsy showed it to be due part to a diaphragmatic abscess. The history of the case was one of influenza.

According to Ricketts and Dick the bacillus of Pfeiffer enters the blood streams and in that way may be carried to any part of the body, where a suitable field is found for propagation, thus becoming the foci for active inflammation, the striking examples of which are endocarditis, arthritis, etc. Infarcts into kidney or liver may also occur, resulting in renal and perirenal abscess and hepatic abscess. Of course, sub-phrenic abscess may occur from the influenza infections, but only in an indirect and indefinite manner, so that we cannot say that the influenza bacilli are really ever productive of sub-phrenic abscess per se.

Other surgical complications may occur, which we will not here mention, but which may be left to be discussed by others.

We are certain from a study of the symptomatology and pathology of influenza that the susceptibility to lesions of the appendix or appendicitis is greatly increased or may be increased directly by bacillus of Pfeiffer. Some authors state that such is true.

Some authors claim that the gastro-intestinal tract is the atrium of the bacillus of Pfeiffer. Whether that is true or not we are assured that hemorrhages may occur from the stomach and intestinal tract due to marked inflammation of the mucosa and the direct result of infection by the said bacilli and it is not improbable that some cases have gastric and duodenal ulcer primarily owe their origin to grippal infection. Inflammation of the cystic and common duct may occur following la grippe, thus producing a catarrhal jaundice and secondarily a cholecystitis, finally lapsing into gall stone or empyema of the gall bladder demanding surgical relief.

